# Social distancing and mask-wearing could avoid recurrent stay-at-home restrictions during COVID-19 respiratory pandemic in New York City

**DOI:** 10.1038/s41598-022-13310-1

**Published:** 2022-06-20

**Authors:** Hae-Young Kim, Anna Bershteyn, Jessica B. McGillen, Jaimie Shaff, Julia Sisti, Charles Ko, Radhika Wikramanayake, Remle Newton-Dame, R. Scott Braithwaite

**Affiliations:** 1 Department of Population Health, New York University Grossman School of Medicine, 227 E 30th Street, New York, NY USA; 2 New York City Department of Health and Mental Hygiene, New York, NY USA; 3 New York City Health and Hospitals, New York, NY USA

**Keywords:** Viral infection, Translational research

## Abstract

Stay-at-home restrictions such as closure of non-essential businesses were effective at reducing SARS-CoV-2 transmission in New York City (NYC) in the spring of 2020. Relaxation of these restrictions was desirable for resuming economic and social activities, but could only occur in conjunction with measures to mitigate the expected resurgence of new infections, in particular social distancing and mask-wearing. We projected the impact of individuals’ adherence to social distancing and mask-wearing on the duration, frequency, and recurrence of stay-at-home restrictions in NYC. We applied a stochastic discrete time-series model to simulate community transmission and household secondary transmission in NYC. The model was calibrated to hospitalizations, ICU admissions, and COVID-attributable deaths over March–July 2020 after accounting for the distribution of age and chronic health conditions in NYC. We projected daily new infections and hospitalizations up to May 31, 2021 under the different levels of adherence to social distancing and mask-wearing after relaxation of stay-at-home restrictions. We assumed that the relaxation of stay-at-home policies would occur in the context of adaptive reopening, where a new hospitalization rate of ≥ 2 per 100,000 residents would trigger reinstatement of stay-at-home restrictions while a new hospitalization rate of ≤ 0.8 per 100,000 residents would trigger relaxation of stay-at-home restrictions. Without social distancing and mask-wearing, simulated relaxation of stay-at-home restrictions led to epidemic resurgence and necessary reinstatement of stay-at-home restrictions within 42 days. NYC would have stayed fully open for 26% of the time until May 31, 2021, alternating reinstatement and relaxation of stay-at-home restrictions in four cycles. At a low (50%) level of adherence to mask-wearing, NYC would have needed to implement stay-at-home restrictions between 8% and 32% of the time depending on individual adherence to social distancing. At moderate to high levels of adherence to mask-wearing without social distancing, NYC would have needed to implement stay-at-home restrictions. In threshold analyses, avoiding reinstatement of stay-at-home restrictions required a minimum of 60% adherence to mask-wearing at 50% adherence to social distancing. With low adherence to mask-wearing and social distancing, reinstatement of stay-at-home restrictions in NYC was inevitable. High levels of adherence to social distancing and mask-wearing could have attributed to avoiding recurrent surges without reinstatement of stay-at-home restrictions.

## Introduction

In March 2020, New York City (NYC) became the epicenter of the COVID-19 epidemic, accounting for more than one-third of the total confirmed cases in the US. Two weeks after the first case was reported in NYC, Governor Cuomo declared stay-at-home restrictions that included banning large gatherings and closing schools and non-essential businesses^1,2^. Facial mask-wearing in public became mandatory in mid-April^3^. Following the introduction of these policies, the daily reported new cases in NYC declined to fewer than 700 per day in early June, down from over 10,000 cases per day at the peak of the epidemic in April 2020.

New York State (NYS) implemented tiered reopening guidance between early June and late September, first reopening industries such as construction and manufacturing, followed by in-store retail, outdoor and indoor dining, and elementary schools, all at limited capacity^4^. However, due to a concern for a potential resurgence of cases, the reopening in each region of the state was contingent on meeting several criteria, including maintaining < 2 new daily hospitalizations per 100,000 residents, and > 30% hospital and ICU bed capacity region-wide. The state-wide policy required ongoing monitoring of these metrics, and recommended the reinstatement of restrictions in a given region if the criteria were exceeded^5^.

Mask-wearing and social distancing are the two critical measures to mitigate the transmission of respiratory diseases^5,6^. Two meta-analyses of respiratory diseases caused by coronaviruses, including Severe acute respiratory syndrome (SARS), Middle East respiratory syndrome (MERS), and SARS-CoV-2, demonstrated that mask-wearing and social distancing effectively reduced viral transmission^7,8^. Using a natural experiment examining the association between the state-wide mandate orders for face cover in public and the daily confirmed COVID-19 cases from all states, a study reported that community-wide mask-wearing could have accounted for declines in COVID-19 growth rates in the US during Spring 2020^9^. Mathematical modeling of SARS-CoV-2 in New York and elsewhere have estimated the extent to which mask-wearing reduces population-level transmission^10^ but has not examined the impact in the context of reinstatement or relaxation of stay-at-home restrictions upon reopening. High adherence to mask-wearing and social distancing may allow the city to reopen by controlling the epidemic, and avoiding surges in cases and hospitalizations thus consequential reinstatement of stay-at-home restrictions.

In this study, we used mathematical modeling to examine the impact of individual adherence to social distancing and mask-wearing on COVID-19 epidemic and duration, frequency, and recurrence of stay-at-home restrictions in NYC. The study findings can have important implications for settings with limited vaccine availability or other emerging infectious diseases in the period when only non-pharmaceutical interventions are available.

## Methods

### Mathematical model

We adapted an existing stochastic, discrete-time model of community transmission of SARS-CoV-2^11,12^ by adding a lagged transmission of community-acquired infections to household members and a compartment to represent quarantine of those who have received testing or contact tracing. We measured outcomes of infections, hospitalizations, and deaths as well as duration, frequency, and recurrence of stay-at-home restrictions by May 31, 2021. The revised model structure has been presented elsewhere^12^. Briefly, the model represents individuals who are susceptible (S), exposed (E), infectious (I), quarantined (Q), or removed/recovered (R).

The model structures are described as following:

$$S_{t} = S_{t - 1} - S_{t - 1} \left( {\beta I_{t - 1} + \beta_{m} Q_{t - 1} + z_{t - 1} } \right)\varepsilon_{t}$$

$$E_{t} = S_{t - 1} \left( {\beta I_{t - 1} + \beta_{m} Q_{t - 1} + z_{t - 1} } \right)\varepsilon_{t } + \left( {1 - \frac{1}{{\delta_{e} }}} \right)E_{t - 1}$$

$$I_{t} = \frac{1}{{\delta_{e} }}E_{t - 1} + \left( {1 - \frac{1}{{\delta_{i} }} + \left[ {\frac{{{\rho }_{{1{ }}} \left( {\delta_{1} - \delta_{{\text{i}}} } \right)}}{{\delta_{1} \delta i}} + \frac{{{\rho }_{{2{ }}} \left( {\delta_{2} - \delta_{{\text{i}}} } \right)}}{{\delta_{2} \delta i}} + { }\frac{{\rho_{3} (\delta_{3} - \delta_{i} )}}{{\delta_{3} \delta_{i} }}} \right]} \right)I_{t - 1}$$

$$Q_{t} = \left[ {\frac{1}{{\delta_{1} }}{\rho }_{1} + \frac{1}{{\delta_{2} }}{\rho }_{2} + { } \frac{1}{{\delta_{3} }}{ }{\rho }_{3} } \right]I_{t - 1} + \left[ {1 - \left( {\frac{1}{{\delta_{i} - \delta_{1} }}\left( {\frac{{\rho_{1} }}{P}} \right)_{ } + \frac{1}{{\delta_{i} - \delta_{2} }}\left( {\frac{{\rho_{2} }}{P}} \right) + { }\frac{1}{{\delta_{i} - \delta_{3} }}\frac{{\rho_{3} }}{P}} \right)} \right]Q_{t - 1}$$

$$R_{t} = \frac{1}{{\delta_{i} }}\left[ {1 - (\rho_{1} + \rho_{2} + \rho_{3} )} \right]I_{t - 1} + \left( {\frac{1}{{\delta_{i} - \delta_{1} }}\left( {\frac{{\rho_{1} }}{P}} \right)_{ } + \frac{1}{{\delta_{i} - \delta_{2} }}\left( {\frac{{\rho_{2} }}{P}} \right) + { }\frac{1}{{\delta_{i} - \delta_{3} }}\frac{{\rho_{3} }}{P}} \right)Q_{t - 1}$$

$$C_{t} \sim Binomial\left( {I_{t} , p} \right)$$

where $${\uprho }_{3} {\text{ is min}}\left( {1, \frac{\lambda *\kappa }{\mu }} \right)*\left( {\theta - min\left( {\rho_{1} + \rho_{2} , \theta } \right)} \right)$$ and $$P\ \text{ is}\ \rho_{1} + \rho_{2} + \rho_{3}$$. All parameters are described in Table 1. In NYC, *z*_t_ is the number of importations on day *t*, which was set to 7 on January 15th and 7 on February 22nd for the importations linked to the initial outbreak in Wuhan and other global importations, respectively, and one to represent the initial case found in NYC. The transmission in the model was stochastic with a log-normal distribution with a standard deviation of 0.722 ($$p)$$, a value determined based on Seattle’s 2018–2019 influenza season^11^.

**Table 1:** Key parameters for the SEIQ-R model with concurrent testing, tracing, and quarantine and disease progression in NYC.

Symbol	Description	Baseline value	References
**Epidemic**			
$\delta_{e}$	Time from exposure to being infectious, days	4.0	^13,14^
$\delta_{i}$	Infectious period, days	8.0	^13,14^
$\beta$	Attack rate at the beginning of the outbreak	Derived	^15^
$\beta_{m}$	Attack rate under quarantine	Derived	^15^
**Index case testing**			
$\delta_{1}$	Time from exposure to index case testing, days*	4.0	Assumption
$\rho_{1}$	Proportion of infected individuals receiving index case testing	0.4	Assumption
**Contact tracing**			
$\delta_{2}$	Time from exposure to contact tracing, days*	4.0	Assumption
$\rho_{2}$	Proportion of contacts of index cases being traced	Derived	
$\theta$	Proportion of population traceable	0.70	Assumption
$\kappa$	Number of contact tracers	3000	^16,17^
$\lambda$	Number of contacts successfully traced per tracer per day	2	^16,17^
*C*_*0*_	Number of contacts per index case before stay-at-home restrictions are imposed	52	^18^
*C*_*1*_	Number of contacts per index case after stay-at-home restrictions are imposed	5	^18^
$\mu$	Number of contacts who need to be traced	Derived	
**Recurrent and random testing**			
$\delta_{3}$	Time from exposure to recurrent and random testing, days	4.0	Assumption
$\rho_{3}$	Proportion of infected individuals receiving recurrent and random testing	Derived	

*This assumes that index cases and contacts will be isolated or quarantined upon receiving the diagnostic and/or laboratory test, whichever comes first.

In addition, we assumed a secondary attack rate of 25% for within-household transmission after March 17, 2020 when stay-at-home policies were instated^19^. The model accounted for age-specific and comorbidity-adjusted infection-fatality ratio (IFR). We calculated the age-specific IFR adjusting for the increased severity for hospitalizations and deaths among those with underlying comorbidities by age groups (10–19; 20–29; 30–39; 40–49; 50–59; 60–69; 70–79; 80 + years). IFR was inferred from the case-fatality ratio in South Korea by mid-March 2020 (1.01%). Given that the testing and contact tracing were widely conducted in Korea, we assumed that CFR would provide a reasonable approximation for IFR. By early March, ~ 95% of the contacts of the confirmed index cases related to the main outbreak cluster in Daegu, Korea, which accounted for > 79% all confirmed cases in Korea, were traced and tested^20^. Therefore, we assumed the IFR would be slightly lower by 5%, resulting in the overall IFR estimate of 0.94. Then, we estimated the IFR in NYC adjusting for the age and comorbidities distribution in NYC. The model was implemented in Python 3.7 and outputs were analyzed and graphed using R 3.6.1.

### Model assumptions and calibration

The model used NYC’s distribution of age and chronic condition, obtained from New York Behavioral Risk Factor Surveillance System Data in 2017^21^ and 2013–2014 New York City Health and Nutrition Examination Survey (NYC HANES)^22^, and census^23^ to estimate the impact of the epidemic on COVID-19 hospitalization and mortality. We assumed that the average time from infection to symptom onset to be 5.1 days^24^, and from symptom onset to hospitalization to be 11 days^25^. Based on the information provided by the NYC Department of Health and Mental Hygiene (DOHMH) during the early epidemic by April 2020, the average lengths of stay at hospitals among non-intensive care unit (ICU) admitted patients were assumed to be 11 days. Critically ill patients were assumed to be first admitted to non-ICU hospital beds for three days, then transferred to the ICU for 21 days before returning to non-ICU hospital beds for another 14 days. Given the absence of effective interventions in the early epidemic, we assumed that 28.8% of the hospitalized would die, with an average length of ICU stay of 2 days before death^25^. In addition, the model was conducted in the context of no available vaccinations, given the timeline of vaccine development. Table 2 shows the list of parameters with uncertainty ranges. In sensitivity analysis, we varied the key parameters across the uncertainty ranges and measured the cumulative deaths (Figure S1).

**Table 2:** Key parameters for the epidemic, clinical outcomes, and disease progression of COVID-19 in NYC.

Description	Baseline value*	Range	References
**Epidemic and clinical outcomes (%)**			
Asymptomatic infections	60.0%	40.50–87.1	^26–28^
Symptomatic infections	40.0%	12.9–59.5	^26–28^
Mild symptoms	73.5%	55.0–91.9	^27,29,30^
Severe symptoms	16.0%	4.9–27.1	^27,29,30^
Critically ill patients admitted to hospitals	9.0%	2.8–15.2	^27,29,30^
Critically ill patients outside hospitals	1.5%	0.5–2.5	^27,29,30^
ICU admission among the hospitalized	36.0%	14.2–45.0	^27,29–32^
Deaths among critically ill patients admitted to ICU	80.0%	60.0–100.0	^27,29–31,33^
Time to disease progression and hospitalization (days)*			
Time to symptom onset (incubation period)	5.1	4.5–5.8	^24,34^
Time from symptom onset to hospitalization	11.0	3.0–13.75	
Time from symptom onset to ICU hospitalization	14.0	4.0–17.5	^31^
Time from symptom onset to death	16.0	0–27.0	^30^
Time in hospitalization for severely ill patients	11.0	3.0–13.5	^35^
ICU length of stays among survivors who were critically ill	21.0	3.0–23.0	^31,36^
ICU length of stays among non-survivors who were critically ill	2.0	0–13.0	^31,36^
Time in non-ICU units after discharged from ICU	14.0	11.6–47.2	^31,36^

*The baseline values were primarily informed by the internal data from NYC DoHMH during the early epidemic by April 2020 when available.

*COVID-19* coronavirus disease-2019, *ICU* intensive care unit, *DOHMH* Department of Health and Mental Hygiene.

The model was calibrated to the publicly available data on daily new hospitalizations for COVID-19 and confirmed and probable data COVID-19 attributable deaths from the NYC DOHMH and the internal data on daily number of patients with COVID-19 in ICUs from the NYS Department of Health’s Hospital Emergency Response Data System (HERDS). The data for HERDS data was self-reported by hospitals. An effective reproduction number (R_e_) was adjusted to minimize the sum of squared residuals between these data and their corresponding model outputs. The daily hospitalization data in mid-April was updated later with more certainty, and this update increased reported hospitalizations by 30% in the two months following the initial report. Therefore, when calibrating the model, we raised the upper 95% confidence interval to allow for up to 30% under-reporting of daily new hospitalizations and number of patients with COVID-19 in ICUs. New daily cases of COVID-19 were not used as a target for calibration due to changing testing eligibility and accessibility throughout the period of analysis. However, the calibration was constrained to ensure that new infections in the simulation must exceed the number of new cases identified through testing.

We estimated the initial reproduction number (R_0_) at the beginning of the epidemic in NYC and the R_e_ after the pandemic peak of March–April 2020. The estimated R_e_ under subsequent relaxation of stay-at-home restrictions was obtained by propagating the uncertainty in the initial R_0_ while maintaining fixed benchmarks for possible levels of mask-wearing and social distancing. On June 1, 2020, NYC launched a new contact-tracing program, *NYC Test & Trace Corps*, hiring 3,000 contact tracers and set a target of carrying out 50,000 daily tests^16,17,37^. Based on these data, we assumed that a citywide random daily testing of 50,000 and contact tracing program with 3000 tracers from June 1, 2020 to May 31, 2021. We assumed that each tracer could successfully trace two contacts of an index case per day, and that contacts were quarantined for an average of four days after being infectious (i.e., half of the average infectious period of eight days). All methods were performed in accordance with the relevant guidelines and regulations.

### Model scenarios for adherence to social distancing and mask-wearing

We simulated model scenarios in which relaxation of stay-at-home restrictions occurred with different degrees of individual adherence to social distancing and mask-wearing. At one extreme, we simulated a return to pre-pandemic activity with no social distancing or mask-wearing. Social distancing was represented as keeping 6 feet of distance from others. We applied the effect of inter-person spacing on SARS-CoV-2 transmission from a meta-analysis of close contact events^8^ where the risk of transmission from an infected individual was reduced by 2.02 times per additional 1 m distance. Social distancing scenarios assumed that 50% of population would maintain 6 feet of distance with all contacts outside the household. We further assumed four different levels of adherence to mask-wearing. Two recent meta-analyses estimated that wearing any face masks including surgical masks, 12–16 layer cotton masks, or N95 respirators reduces transmission by 65–85%^7,8^. We conservatively used the lower estimate of 65% as a benchmark for mask efficacy. This was further modified by the estimated proportion of individuals wearing masks outside the home, estimated at between 65% and 89% in surveys in NYC and elsewhere^38–41^. Given that the impact of mask-wearing would greatly vary by population coverage and adherence, we varied the percentage of adherence to wearing any mask correctly at 50%, 70%, and 90% to represent low, moderate, and high adherence^38–41^.

### Thresholds to reinstate or relax stay-at-home restrictions

We incorporated the guidelines from NYS health authorities for the conditions under which regional stay-at-home restrictions could be relaxed or needed to be reinstated^5^. We assumed that stay-at-home restrictions would resume if the rate of new COVID-19 hospitalizations exceeded 2 per 100,000 residents per day. In consultation with NYC DOHMH, we developed the assumption that stay-at-home restrictions may be relaxed if COVID-19 hospitalizations dropped to the lowest levels observed prior to the initial relaxation on June 1, 2020: a rate of 0.8 per 100,000 residents per day. We assumed that stay-at-home restrictions and relaxations would continue to occur in cycles until May 31, 2021, if NYC meets the thresholds of ≥ 2 or ≤ 0.8 hospitalizations per 100,000 residents per day to relax or reinstate stay-at-home restrictions, respectively. We assumed phased relaxation over 4 weeks as done in Summer 2020, with a multiplicative effect on R_e_ for each stage of relaxation, resulting in exponential growth in R_e_ between the fully-restricted state and the fully-relaxed state.

### Ethics approval

This study used publicly available data and was exempt from ethical review by the Institutional Review Boards of both New York University Grossman School of Medicine and NYC DOHMH.

## Results

Model calibration to the growth in hospitalizations, ICU admissions, and deaths early in the NYC SARS-CoV-2 epidemic yielded an estimated R_0_ of 3.08 (IQR 2.97–3.42) at the beginning of the epidemic in NYC with an R_e_ that declined to a minimum value of 0.50 (IQR 0.47–0.59) on April 10. Stay-at-home restrictions such as the closure of non-essential businesses and schools, large in-person gatherings, as well as individual adherence to social distancing and mask-wearing, succeeded in bringing the R_e_ below one until summer 2020. The R_e_ was estimated to rebound upon relaxation of stay-at-home restrictions depending on different adherence levels to social distancing and mask-wearing (Table 3).

**Table 3:** Estimated effective reproduction numbers (R_t_) after full reopening under the different adherence to social distancing and mask-wearing.

Scenario	Adherence to social distancing*	Adherence to mask-wearing	R_e_ in full reopening (IQR)
1	None	None	3.080 (2.908, 3.633)
2	50%	None	1.957 (1.848, 2.308)
3	None	50%	2.079 (1.963, 2.452)
4	50%	50%	1.321 (1.247, 1.558)
5	None	70%	1.679 (1.585, 1.980)
6	50%	70%	1.067 (1.007, 1.258)
7	None	90%	1.278 (1.207, 1.508)
8	50%	90%	0.812 (0.767, 0.958)

*Social distancing is equivalent to keeping 6 feet distance from others.

*IQR* Interquartile Range.

**Figure 1:**
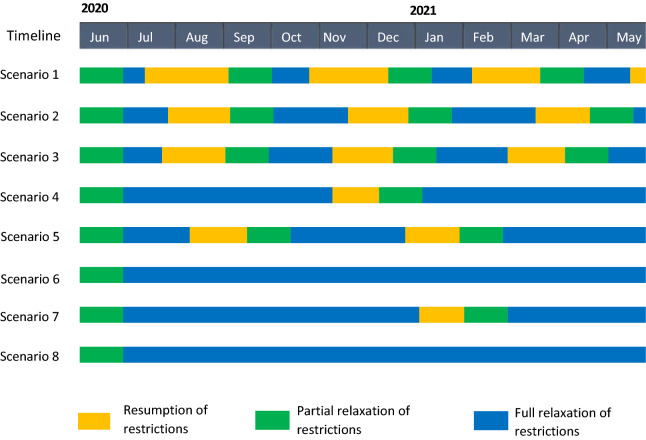
Estimated time spent under resumption and relaxation of restrictions between June 1, 2020 and May 31, 2021 by adherence to social distancing and mask-wearing. Yellow bars indicate the time spent under resumption of restrictions. Green and blue bars indicate the time up to 4 weeks (partial relaxation) and ≥ 4 weeks (full relaxation) after relaxation of restrictions, respectively. Adherence to mask-wearing is assumed as follows: 0% (Scenario 1 and 2), 50% (Scenario 3 and 4), 70% (Scenario 5 and 6), or 90% (Scenario 7 and 8). Adherence to social distancing is assumed to be 0% (Scenario 1, 3, 5, and 7) or 50% (Scenario 2, 4, 6, and 8).

**Figure 2:**
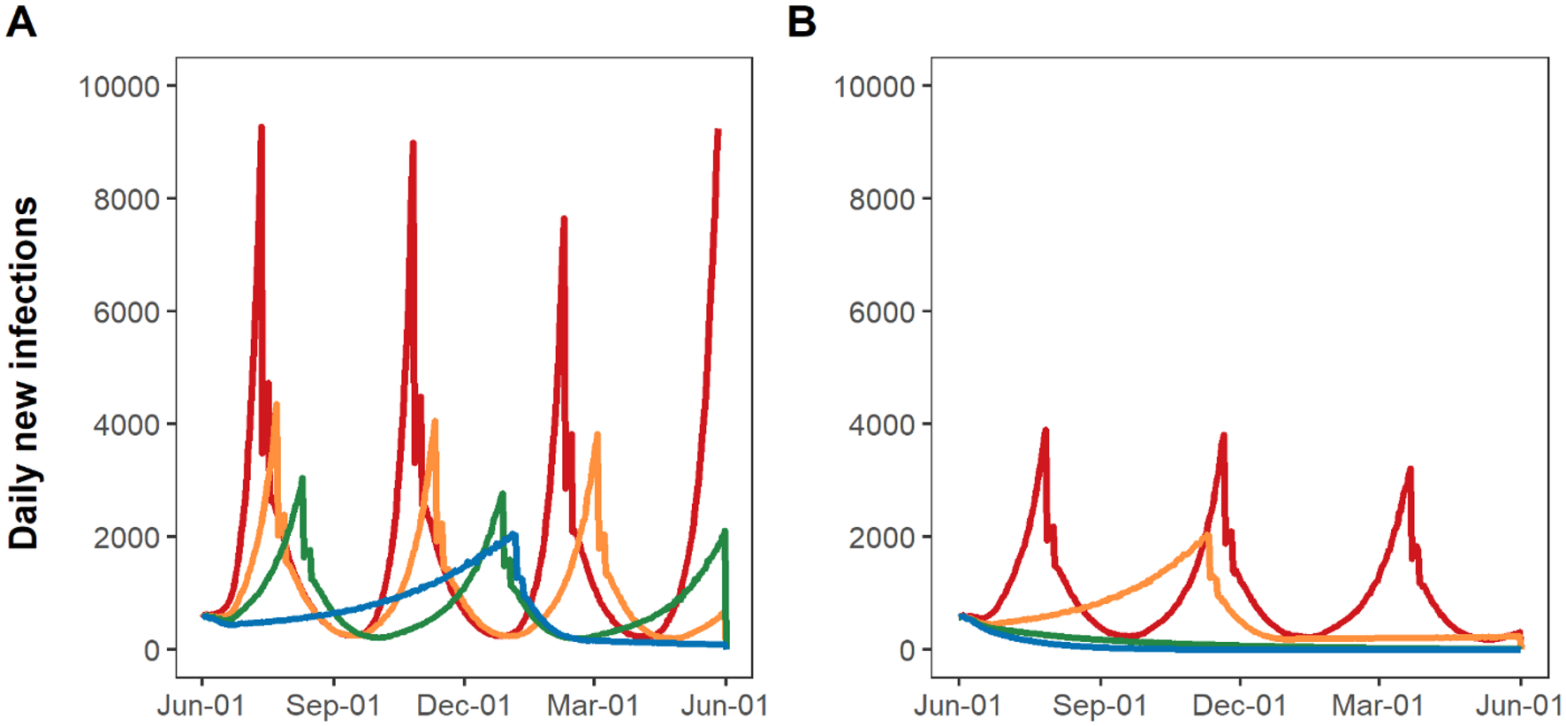
Daily COVID-19 new infections under resumption and relaxation of restrictions between June 2020 and May 2021: (**A**) without adherence to social distancing and (**B**) with 50% adherence to social distancing. Each colored line indicates the level of mask adherence at 0% (red), 50% (orange), 70% (green) or 90% (blue).

**Figure 3:**
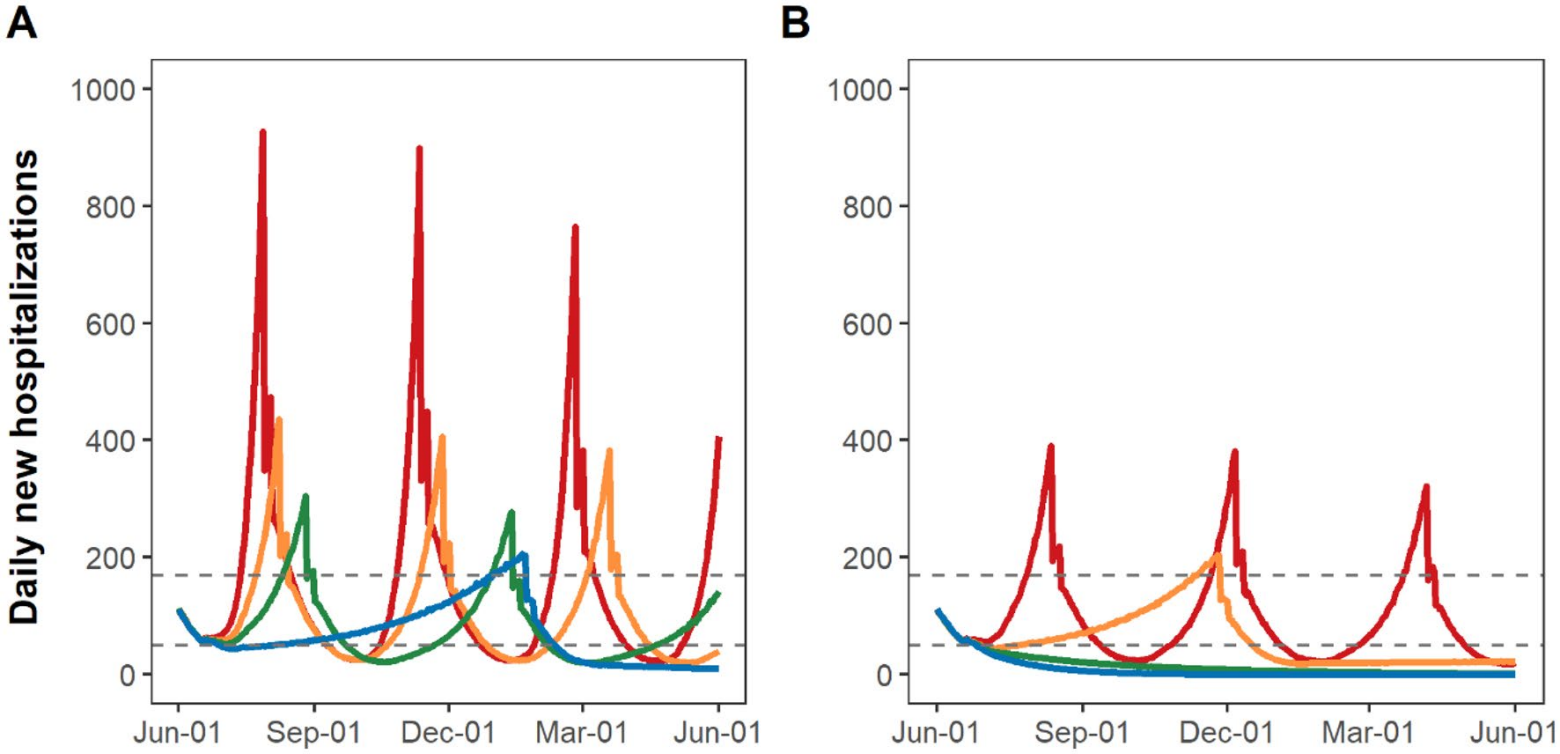
Daily COVID-19 new hospitalizations under resumption and relaxation of restrictions between June 2020 and May 2021: (**A**) without adherence to social distancing and (**B**) with 50% adherence to social distancing. Each colored line indicates the level of mask adherence at 0% (red), 50% (orange), 70% (green) or 90% (blue).

Reinstatement of stay-at-home restrictions would immediately lower transmission (Fig. 1); however, due to the time lag from infections to hospitalizations, daily hospitalizations would continue to grow, peaking ~16 days after the reinstatement of stay-at-home restriction (Fig. 2). Similarly, deaths would continue to grow and peak 21 days after the resumption of stay-at-home restrictions (Fig. 3).

### Impact of adherence to social distancing and mask-wearing on epidemic growth upon reopening, and duration and frequency of reinstatement of stay-at-home restrictions

**Figure 4:**
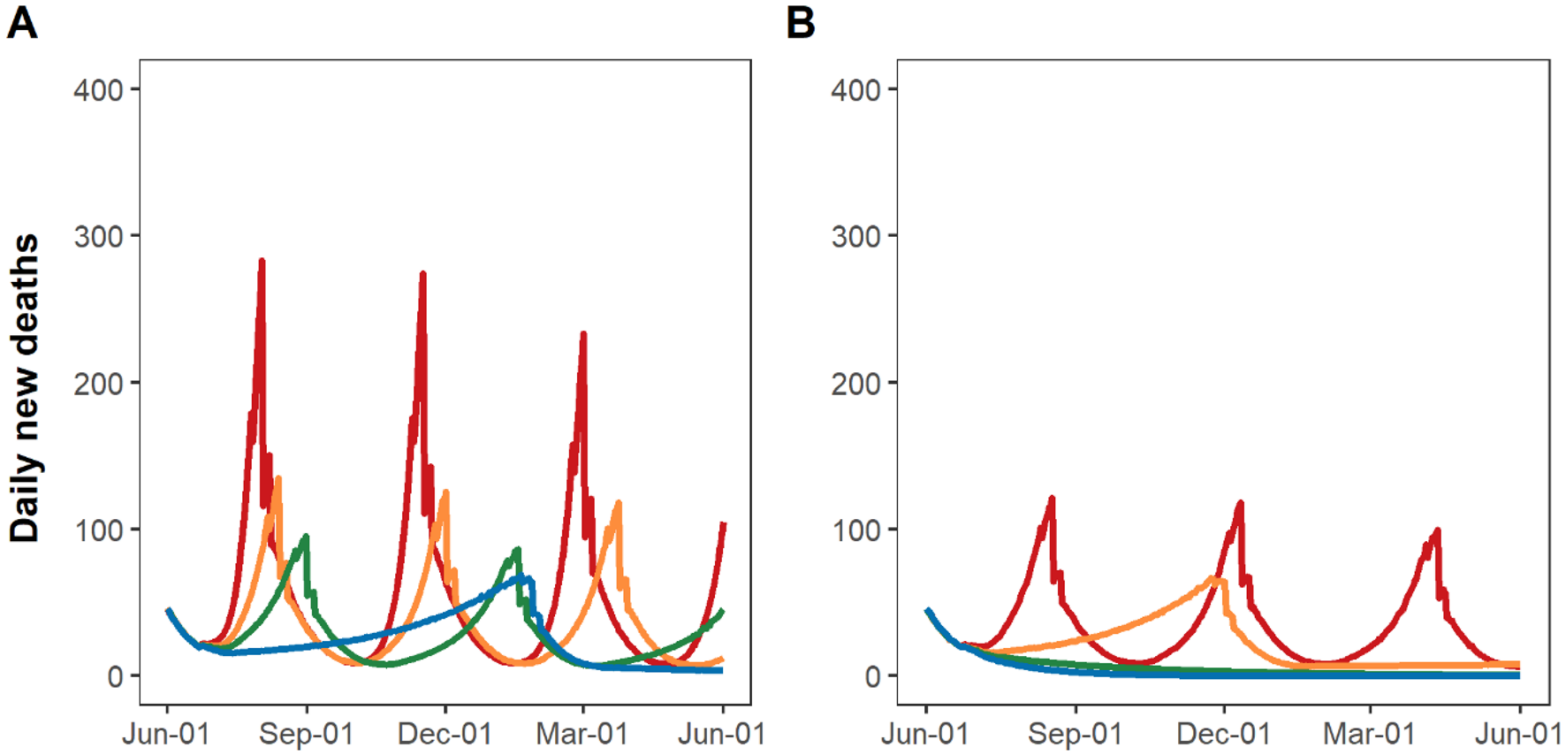
Daily COVID-19-attributable deaths under resumption and relaxation of restrictions between June 2020 and May 2021: (**A**) without adherence to social distancing and (**B**) with 50% adherence to social distancing. Each colored line indicates the level of mask adherence at 0% (red), 50% (orange), 70% (green) or 90% (blue).

Under the most pessimistic assumptions of no mask-wearing and no social distancing (Scenario 1), NYC would have cycled between reinstatement and relaxation of stay-at-home restrictions four times by May 31, 2021. Only 26% of that time period would have been spent under full relaxation of stay-at-home restrictions (Fig. 1; Scenario 1, blue bars), with an additional 31% spent under partial relaxation (green bars) and 43% spent under stay-at-home restrictions (yellow bars). Under this assumption, the epidemic would have grown the fastest, reaching 9,300 daily new infections (Fig. 2A, red curve). A 54-day period of reinstated stay-at-home restrictions would have been necessary to bring new hospitalizations below the re-opening threshold of 0.8 per 100,000 residents (Fig. 3A, red curve). Phased reopening would have lasted 52 days before reaching the closure threshold, and the next closure would have lasted for 51 days before again reaching the re-opening threshold. More COVID-19 deaths (n = 18,690) would have occurred over the four cycles of re-opening than the cumulative COVID-19 deaths that had occurred (n = 17,716) prior to June 2020 (Fig. 4A, red curve).

**Figure 5:**
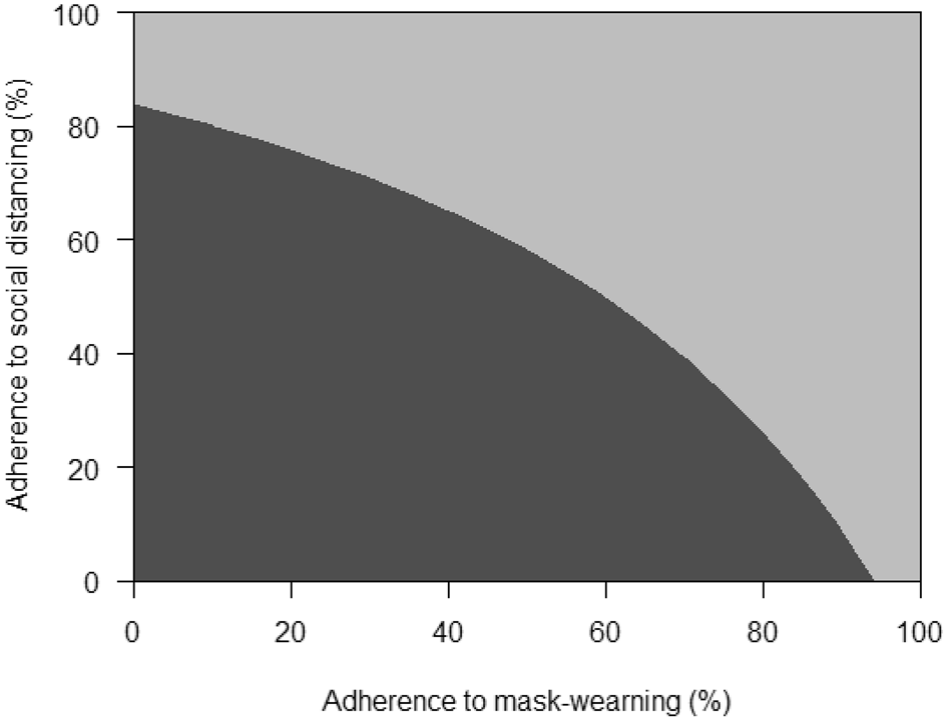
Threshold analysis of adherence necessary to prevent any reinstatement of stay-at-home restrictions between June 2020 and May 2021 in NYC. Dark grey region corresponds to a range of adherence to social distancing and mask-wearing which necessitates the reinstatement of stay-at-home restrictions, while light grey region corresponds to a range avoiding any reinstatement of stay-at-home restrictions.

With the addition of either 50% adherence to social distancing (Scenario 2) or low adherence to mask-wearing (Scenario 3), the epidemic would have grown more slowly upon relaxation of stay-at-home restrictions and required reinstatements of restriction three times by May 31, 2021, increasing the portion of time spent under full relaxation of stay-at-home restrictions from 26 to 37%. Low adherence to mask-wearing with 50% adherence to social distancing (Scenario 4) or high adherence to mask-wearing without social distancing (Scenario 7) would have required one-time reinstatement of stay-at-home restrictions by May 31, 2021 (Fig. 1), further increasing the portion of time spent under full relaxation of stay-at-home restrictions to 76%. The combination of 50% adherence to social distancing and moderate to high adherence to mask-wearing would have enabled NYC to remain fully open without any reinstatement of stay-at-home restrictions (Scenarios 6 and 8). In threshold analyses (Fig. 5), avoiding reinstatement of stay-at-home restrictions required a minimum of 60% adherence to mask-wearing together with 50% adherence to social distancing.

## Discussion

NYC succeeded in reducing SARS-CoV-2 transmission through a stay-at-home order and restrictions on non-essential businesses, schools, and other in-person gatherings. As transmission decreased, phased reopening began in June 2020. However, there was a need to understand the potential impacts of reopening and identify opportunities to reduce harm. Our modeling results indicate that low adherence to mask-wearing and social distancing would have led to undesired consequences of repeated cycles of shutdown and reopening. On the other hand, our modeling results also highlight that high adherence to social distancing and mask-wearing could prevent any subsequent cycles of shutdown and reopening.

After the relaxation of stay-at-home restrictions, cities, states, and countries across the globe experienced resurgence^42–44^. Multiple states in the United States and countries across the globe paused reopening plans or reinstated restrictions. As cases rapidly surged, UK reinstated a national lockdown in early November 2020^45^, and other countries including Greece and Germany subsequently reinstated and extended lockdown measures^46,47^. In contrast, countries including Hong Kong and Vietnam recorded fewer than 2,000 cases per million and 30 deaths per million by the end of 2021^42^. These countries implemented highly restrictive mitigation measures and demonstrated high adherence to mask-wearing and social distancing, which likely contributed to their low COVID-19 burden and ability to resume many social, economic, educational, and religious activities^48–50^.

In NYC, adherence to mask-wearing was moderate, varying from 65% to 89% in the summer of 2020^38–41^. At these levels of mask-wearing, our model projected that NYC would have exceeded the threshold to reinstate stay-at-home restrictions if people were not successfully able to maintain social distancing. Prior to citywide metrics exceeding thresholds, NYC and NYS deployed response programs in particular neighborhoods experiencing growing caseloads including targeted restrictions^51^. These targeted stay-at-home restrictions likely contributed to slowing down the epidemic growth in these selected neighborhoods^52^.

The availability and uptake of SARS-CoV-2 testing, the ability to conduct contact tracing services, and access to opportunities for safe isolation are key elements of epidemic control. We have previously shown^12^ that NYC’s testing and contact tracing capacities contributed to reducing transmission, but the magnitude of this effect would be insufficient to contain the epidemic if mask-wearing and social distancing were not also practiced.

Our study has several limitations. First, we represented a simplification of the impact of social distancing and mask wearing, and did not incorporate differences in these behaviors over or by neighborhood. Neighborhoods with more crowded living conditions and greater proportions of essential workers may experience differential impacts of masks and social distancing. Indeed, a study showed that higher levels of COVID-19 infections were observed in the neighborhoods where subway usage declined the least, in part due to the concentration of essential workers in these communities^53^. Second, the compartmental model used in this analysis did not capture social networks, geographic variation, or individual-level variation such as the propensity for superspreading. The model did represent household transmission during lockdown with a simple assumption that 25% of community cases would beget a household case after one generation time, but actual household transmission is more complex and heterogeneous. Third, we have not directly measured age-mixing patterns and did not attempt to predict possible shifts in the age-distribution of infections, which may have significant effects on disease burden^54^. Fourth, important aspects of the interaction between the impact of testing and contact tracing and the impact of social distancing and mask wearing remain unexplored. However, our previous work suggests that current levels of testing and contact tracing would be far from sufficient for avoiding epidemic resurgence^12^ unless paired with mask-wearing and social distancing, although innovations such as daily point-of-use testing^55^, automated digital contact tracing^56–58^, and targeted testing through sewage surveillance^59,60^ could amplify the impact of testing and tracing.

Our work was conducted prior to the availability of vaccines or other pharmaceutical interventions against COVID-19. On December 13th, 2020, a nurse in NYC became the first American to become vaccinated against COVID-19 outside of experimental trials. As vaccines became more broadly available, thresholds for imposing stay-at-home orders ceased to be followed. As of March 2022, 97% of NYC adults have received at least one vaccine dose^27^, and an adaptive reopening approach is unlikely, provided that vaccines maintain efficacy against SARS-CoV-2 variants. However, our analysis may have relevance in settings with limited vaccine availability, in the event of a future variant that escapes vaccine protection, or as a framework for planning public health responses to future pandemics.

## Conclusions

Our modeling suggests that a combination of social distancing with moderate to high levels of mask-wearing could avoid the need for reinstatement of stay-at-home restrictions when vaccination and effective treatments for SARS CoV-2 are unavailable. Even low to moderate adherence may suffice to enable relaxation of stay-at-home restrictions for the majority of time. However, low adherence to mask-wearing and social distancing, even in the context of testing and contact tracing at feasible levels, would have led to multiple cycles of reinstated stay-at-home restrictions, with a minority of time spent fully reopened.

## Supplementary Information

Supplementary Information.

## Data Availability

Data on COVID-19 cases, hospitalizations, and deaths in NYC is publicly available at https://github.com/nychealth/coronavirus-data by the NYC DOHMH. Data on daily number of patients with COVID-19 in ICUs from the NYS Department of Health’s HERDS were used under the agreement for the current study. However, the data are available from the authors upon reasonable request and with permission of the NYS Department of Health’s HERDS.
