# Supplementary material for: Social distancing and mask-wearing could avoid recurrent stay-at-home restrictions during COVID-19 respiratory pandemic in New York City
Source: Sci Rep. 2022 Jun 20;12:10312. doi: 10.1038/s41598-022-13310-1 (PMC9207433; doi:10.1038/s41598-022-13310-1)
Supplement: Supplementary file 1 — Supplementary Information. [file 41598_2022_13310_MOESM1_ESM.docx]

**Supplementary material**

**Social distancing and mask-wearing could avoid recurrent stay-at-home restrictions during COVID-19 respiratory pandemic in New York City**

We have simulated how cumulative COVID-19 deaths vary as we change the key parameters across its uncertainty range.

**Figure S1.** One-way sensitivity analyses for the key parameters. Bars represent the cumulative COVID-19 deaths between June 1^st^, 2020 and May 31^st^, 2021, under the high (red line) and low (blue line) bounds associated with each parameter, holding all other parameter values constant.
